# Springing of Plates

**Published:** 1853-01

**Authors:** J. Harris


### *Springing of Plates.* By Dr. J. Harris.

Mr. Editor :—The perplexing subject of the springing of plates, in the construction of whole sets, or large blocks of teeth, has elicited, from the pens of different dentists, a variety of theories, upon the causes of such springing, as well as various suggestions for the prevention of it, few of which that I have seen, are altogether satisfactory to me. Whether my speculations upon this subject are worth laying before your readers, I shall leave you to decide.

Our first object should be to discover the cause or causes of our difficulties, and then seek for the remedy. According to my mode of reasoning, the causes of such springing are much more obvious, than a means of entirely preventing it.

In soldering the teeth to the plate, we cover the peripheral portion of it with plaster, sand and the teeth, thus preventing the flame of the lamp from coming in contact with it, whilst the part, within the circle of the teeth, is exposed to a very high temperature. The consequence of heating one portion of the plate to a much higher temperature than the other, must necessarily be a different arrangement of the particles of the gold, so as to accommodate the expanded central portion to the less heated, and therefore less expanded peripheral part of the plate. This new arrangement of the particles of the gold, may result either in a thickening of the central portion of the plate, or in an increase of its convexity, or it may consist of both at the same time. Were the less heated portion glass or other brittle body, as we are all aware, it would crack, but gold being more tenacious in the adhesion of its particles, either bends, stretches, or condenses, according to circumstances.

The particles of the gold having accommodated themselves to the different temperatures of the plate, with the addition of a portion of solder more contractile still than the gold and all confined by the plaster, strengthened probably by wire ; it appears to me very evident that, when it cools, the outer or peripheral part will be drawn inwards, producing the springing of the plate, of which we all, I presume, have more or less unpleasant experience.

Now, in regard to the remedying of our difficulty, I have two means to suggest, in conformity with the causes already alluded to, which promise, in my opinion, at least some security from such accident, namely: to heat the central portion of the plate to a very high temperature, whilst the peripheral part is protected from the flame, previous to swedging it up the last time between the models. This last application of heat to the plate should be as nearly as possible in the same proportion to the different parts of the plate, as that required in soldering. In putting up the piece for soldering, use a large proportion of sand, at least one half, so as to allow the mixture to give before the plate, as it expands in heating.

This last suggestion is predicated upon the principle that as the central part expands the peripheral portion will be bent outwards, and on cooling may return to its original position.

I do not pretend that either of the means of preventing named above, are at all calculated to obviate that part of the springing which is the result of a difference in the contractile qualities of the gold and solder. Neither do I claim to be one of those "fortunate souls" who meet with no difficulties of the kind; yet as I have generally succeeded in springing my plates back so nearly to the right shape by bending as to give satisfaction, without even having damaged a tooth, I feel as if I had some cause for thankfulness to the "good genius" presiding over my success. I seldom connect the back of the teeth with solder. J. HARRIS.

*Salem, O.*, Dec. 1852.

